# GPI-anchored Gas1 protein regulates cytosolic proteostasis in budding yeast

**DOI:** 10.1093/g3journal/jkad263

**Published:** 2024-01-30

**Authors:** Yuhao Wang, Linhao Ruan, Rong Li

**Affiliations:** Center for Cell Dynamics and Department of Cell Biology, Johns Hopkins University School of Medicine, Baltimore, MD 21205, USA; Biochemistry, Cellular and Molecular Biology (BCMB) Graduate Program, Johns Hopkins University School of Medicine, Baltimore, MD 21287, USA; Center for Cell Dynamics and Department of Cell Biology, Johns Hopkins University School of Medicine, Baltimore, MD 21205, USA; Center for Cell Dynamics and Department of Cell Biology, Johns Hopkins University School of Medicine, Baltimore, MD 21205, USA; Mechanobiology Institute and Department of Biological Sciences, National University of Singapore, Singapore 117411, Singapore

**Keywords:** Gas1, GPI, proteostasis, mitochondria, MAGIC, budding yeast

## Abstract

The decline in protein homeostasis (proteostasis) is a hallmark of cellular aging and aging-related diseases. Maintaining a balanced proteostasis requires a complex network of molecular machineries that govern protein synthesis, folding, localization, and degradation. Under proteotoxic stress, misfolded proteins that accumulate in cytosol can be imported into mitochondria for degradation through the “mitochondrial as guardian in cytosol” (MAGIC) pathway. Here, we report an unexpected role of Gas1, a cell wall-bound glycosylphosphatidylinositol (GPI)-anchored β-1,3-glucanosyltransferase in the budding yeast, in differentially regulating MAGIC and ubiquitin-proteasome system (UPS). Deletion of *GAS1* inhibits MAGIC but elevates protein ubiquitination and UPS-mediated protein degradation. Interestingly, we found that the Gas1 protein exhibits mitochondrial localization attributed to its C-terminal GPI anchor signal. But this mitochondria-associated GPI anchor signal is not required for mitochondrial import and degradation of misfolded proteins through MAGIC. By contrast, catalytic inactivation of Gas1 via the *gas1-E161Q* mutation inhibits MAGIC but not its mitochondrial localization. These data suggest that the glucanosyltransferase activity of Gas1 is important for regulating cytosolic proteostasis.

## Introduction

Many aging-related degenerative diseases are associated with loss of proteostasis characterized by protein misfolding and formation of protein aggregates (Balch *et al*. 2008; Labbadia and Morimoto 2015; López-Otín *et al*. 2023). Major protein quality control pathways such as UPS and autophagy play essential roles in clearance and turnover of misfolded proteins (Pohl and Dikic 2019). Recently, an emerging role of mitochondria in proteostasis has been noted (Andréasson *et al*. 2019; Ruan *et al*. 2020). Mitochondria not only contribute to cellular metabolism (Wai and Langer 2016) but also facilitate the clearance of misfolded proteins through a process termed as “mitochondrial as guardian in cytosol” (MAGIC) (Ruan *et al*. 2017; Wang *et al.* 2023). In the budding yeast *Saccharomyces cerevisiae*, misfolded cytosolic proteins can translocate into mitochondria through mitochondrial import channels, and the yeast Lon protease, Pim1, is involved in the degradation of imported misfolded proteins in the mitochondrial matrix, which in turn facilitates the dissolution of cytosolic protein aggregates associated with mitochondria (Ruan *et al*. 2017; Wang *et al*. 2023).

Several lines of evidence indicate that a similar pathway may exist in higher organisms. In human RPE-1 cells, the model unstable protein (FlucSM or FlucDM) but not stable glutathione S-transferase can be imported into mitochondria (Gupta *et al*. 2011; Ruan *et al*. 2017). In HeLa cells, proteasomal inhibition by MG132 can induce the import of unfolded cytosolic GFP into mitochondria through the joint action of mitochondrial outer membrane protein FUNDC1 and cytosolic chaperone HSC70 (Li *et al*. 2019). Many disease-related proteins such as α-synuclein, FUS, and TDP-43 also accumulate in the mitochondria of various human cell models and influence mitochondrial fitness (Devi *et al*. 2008; Wang *et al*. 2016; Wang *et al*. 2023; Deng *et al*. 2018). These findings suggest that MAGIC may be a conserved process that connects cytosolic proteostasis with mitochondrial function.

To elucidate the molecular mechanism and regulation of MAGIC, we conducted an unbiased genetic screen using the non-essential budding yeast knockout library and unveiled potential regulators of MAGIC including *GAS1* (Wang *et al*. 2023). Gas1 (glycophospholipid-anchored surface protein 1) is a β-1,3-glucanosyltransferase anchored to the outer leaflet of the plasma membrane via a GPI moiety and crosslinked with the cell wall (Nuoffer *et al*. 1991; Popolo and Vai 1999; Ragni *et al*. 2007; Yin *et al*. 2007). Gas1 elongates the β-1,3-glucan chains and plays an important role in the expansion and remodeling of the cell wall (Mouyna *et al*. 2000). In a genome-wide analysis of GFP-tagged yeast proteins, it was reported that Gas1-GFP protein localizes to ER, nuclear periphery, and mitochondria (Huh *et al*. 2003). The suspected mitochondrial localization spurred us to investigate the role of *GAS1* in MAGIC in the budding yeast where MAGIC was initially uncovered (Ruan *et al*. 2017).

Here, we show that *GAS1* is a positive regulator of MAGIC, and loss of *GAS1* prevents the accumulation and degradation of misfolded proteins in mitochondria. We also provide insights into the subcellular localization of Gas1-GFP by showing that its C-terminal GPI anchor signal, but not the main part of the protein, localizes to mitochondria. Despite this atypical localization, the catalytic activity of Gas1, but not the mitochondria-associated GPI anchor signal, is required for maintaining MAGIC. The inhibition of MAGIC was also observed, albeit less pronounced, in 2 other cell wall mutants. Moreover, *GAS1*-deficient cells exhibit elevated protein ubiquitination and UPS-mediated protein degradation of an N-end rule substrate (Ub-R-EGFP). Taken together, our work identifies an unexpected role of yeast *GAS1* in regulating proteostasis pathways and suggests a novel connection between cytosolic proteostasis and cell wall integrity.

## Materials and methods

### Yeast strains and growth conditions

Yeast strains used in this study are based on the BY4741 strain background. All yeast strains and relevant plasmids are listed in Supplementary Table 1. Gene deletion and protein tagging were performed by using PCR-mediated homologous recombination and verified by PCR genotyping. At least 3 independent colonies were stored and analyzed for each experiment. *GAS1-GFP* and *MIG1-GFP* strains were obtained from the yeast GFP clone collection (Huh *et al*. 2003). *PUS1-RFP* strain was retrieved from the yeast RFP library (Yofe *et al*. 2016). *bgl2Δ* and *gas5Δ* were retrieved from the non-essential yeast knockout library (Giaever *et al*. 2002). In the FlucSM split-GFP assay (also used in Wang *et al*. 2023), GFP_1-10_ was fused with the mitochondrial matrix protein Grx5 under the *GPD* promoter and stably integrated into the yeast genome at the *TRP1* locus. FlucSM-HA-GFP_11_ (Ruan *et al*. 2017) under the *GAL1* promoter and GEM transcriptional factor (Costa *et al*. 2018) were cloned and stably integrated into the yeast genome. Mitochondria were labeled with Tom70-mCherry in the split-GFP assay or with matrix-targeting MTS-mCherry in other imaging experiments.

To construct *gas1-N528K* and *gas1-E161Q* mutants with a GFP tag, *GAS1-GFP* was cloned and underwent site-directed mutagenesis, before stably integrated into the *HO* locus. To construct *gas1-E161Q* mutants for the yeast spotting assay, split-GFP assay, and imaging of protein aggregates, a plasmid containing *gas1-E161Q* was linearized and integrated into the *KanMX6* locus of the corresponding *gas1Δ* strain. For the dually labeled *mCherry-GAS1-GFP* strain, *mCherry-GAS1* was cloned and integrated into the *HO* locus of *Δgas1* strain, followed by a second transformation to tag the C-terminus of mCherry-Gas1 protein with GFP. The C-terminal 31 residues of Gas1 after the cleavage site N528 were fused with a GFP at the C-terminus to construct the GPI*-GFP, and the ER-targeting signal peptide of Gas1 was added to the N terminus to construct the SS-GPI*-GFP. To replace the GPI* sequence of the endogenous *GAS1*, the GPI anchor signal sequence of *GAS3* (C-terminal 26 residues) or *GAS5* (C-terminal 22 residues) together with a *KanMX6* selective cassette was amplified from corresponding plasmids in the Molecular Barcoded Yeast ORF Library (Ho *et al*. 2009) and inserted into the endogenous *GAS1* locus right after the genomic sequence of N528. Replacements were validated by colony PCR and sequencing. All variants of *GAS1* were expressed under the *GAS1* promoter. *Ub-R-EGFP* under *CUP1* promoter was cloned from the plasmid pYES2-Ub-R-EGFP (Addgene #11953) (Heessen *et al*. 2003) and stably integrated into the *TRP1* locus.

Standard yeast extract-peptone supplemented with 2% (w/v) glucose (YPD) was used for transformations, biochemical analyses, and spotting assays. Synthetic complete (SC) supplemented with 2% (w/v) glucose was used for growing cells for confocal and super-resolution imaging. Yeast cultures and plates were incubated at 30°C except during a 42°C heat shock. Optical density at 600 nm (OD_600_) was used to estimate the amount of yeast cells.

### Drug treatments

β-Estradiol (E2758, MilliporeSigma) was dissolved in ethanol and added to a final concentration of 1 μM for 90 min. Tunicamycin (T7765, MilliporeSigma) was dissolved in DMSO and used at a final concentration of 10 μg/ml for 2 h. DTT (R0861, Thermo Fisher) was dissolved in H_2_O and added into the yeast cell cultures at a final concentration of 10 mM for 2 h. Cycloheximide [CHX (239764, MilliporeSigma)] was dissolved in DMSO, and 100 μg/ml was used to treat cells for the indicated period. Calcofluor white (CFW) (F3543, MilliporeSigma), was dissolved in DMSO to 10 mg/ml as stock and added into the autoclaved YPD media plus 2% (w/v) agar at a final concentration of 10 μg/ml before solidification. CuSO_4_ (C1297, MilliporeSigma) was dissolved in H_2_O and 1 mM was used to treat cells for 30 min.

### Confocal microscopy and imaging conditions

Live cell images were acquired using a Yokogawa CSU-10 spinning disc on the side port of a Carl Zeiss 200 m inverted microscope. Laser 488 or 561 nm excitation was applied to excite GFP or mCherry, respectively, and the emission was collected through the appropriate filters onto a Hamamatsu C9100-13 EMCCD on the spinning disc confocal system. Regarding the multi-track acquisition, the configuration of alternating excitation was used to avoid the bleed-through of GFP. The spinning disc was equipped with a 100 × 1.45 NA Plan-Apochromat objective.

Yeast culture condition for imaging: yeast cells were cultured in SC plus 2% glucose overnight at 30°C. The cells were then refreshed in the corresponding media for at least 3 h at 30°C until reaching an OD_600_ of about 0.3. For the estradiol-GEM inducible system (Costa *et al*. 2018; Wang *et al*. 2023), 1 μM of β-estradiol was added to the media for 90 min at 30°C. All images in the same experiments were acquired with identical laser and exposure settings. For yeast 3D imaging, 0.5 μm step size for a total of 6 μm in the *Z* axis was applied. Image processing was performed using ImageJ software (NIH). For visualization purposes, images were scaled with bilinear interpolation and shown as the middle *Z* panel or maximum intensity projection (MIP) on *Z* for individual or merged fluorescent channels.

### Super-resolution imaging

Structured illumination microscopy (SIM) images were acquired with a GE OMX-SR Super-Resolution Microscope 3D Structure Illumination (3D-SIM) equipped with high-sensitivity PCO sCMOS cameras. GFP and mCherry were excited with 488 and 568 nm lasers, respectively. The SIM images were reconstructed with the Softworx and aligned following the Applied Precision protocols. 3D Rendering was performed with Imaris (Oxford Instruments Group).

### Split-GFP quantification

Split-GFP fluorescence from confocal images was quantified by using a custom Python code described before (Ruan *et al*. 2017; Wang *et al*. 2023). In brief, mCherry and GFP intensities were summed along the *Z* axis and then subjected to a random walk segmentation of the background and watershed segmentation of adjoining cells. For each cell, the mCherry channel was thresholded at 5% of maximal value to detect mitochondria, and median GFP intensity within mitochondria was calculated as the spGFP intensity per cell. Mean spGFP intensities from populations of at least 3 biological repeats were used for the following analyses. Quantifications were shown either as an absolute intensity value with an arbitrary unit (a.u.) or in a relative term to highlight the differences between strains and conditions.

### Yeast whole cell lysis and immunoblots

Yeast cells in the indicated background were collected by centrifugation and snap-frozen in liquid nitrogen for storage. Pellets were disrupted, boiled in 120 μl LDS sample buffer with 40 mM DTT (Thermo) for 10 min, and vortexed with an equal volume of 0.5 mm acid-washed glass beads to break cells at 4°C for 2 min with 1-min intervals. Cell lysates were re-boiled for 5 min, separated from glass beads by 15,000 *g* centrifugation at room temperature for 30 s, and analyzed by SDS-PAGE.

Gel transfer was performed in iBlot2 (Thermo) and immunoblots were developed using Clarity Western ECL substrate (Bio-Rad) for HRP-linked secondary antibodies or directly using fluorescent IRDye secondary antibodies (LI-COR). Images were acquired by using LI-COR imaging system and analyzed in Image Studio (LI-COR). HA-tag (C29F4) rabbit mAb #3724 from Cell Signaling Technology. PGK1 mouse mAb (22C5D8) from Invitrogen. GFP Living Colors A.v. mAb clone JL-8 (632381) from Takara Bio. Ubiquitin mouse mAb (P4D1) from Santa Cruz Biotechnology. Gas1 (N-terminal) antiserum was kindly provided by Dr Hongyi Wu at the Mechanobiology Institute, NUS, Singapore.

### Mig1 nucleocytoplasmic translocation

The nucleocytoplasmic distribution of Mig1-GFP was quantified using a custom ImageJ macro and MATLAB script as reported previously (Kelley and Paschal 2019). Nuclear protein Pus1-RFP was used to create a nucleoplasmic mask for the individual cell. Cytoplasm was defined by a dilated nuclear mask. The nuclear-cytoplasmic ratio of each cell was calculated by dividing the mean nuclear intensity by the mean cytoplasmic intensity. Populational mean nuclear-cytoplasmic ratio of at least 3 biological replicates was used for statistical analyses.

### Yeast spotting assay

Single colonies of wild-type and mutant cells were inoculated in YPD media at 30°C for overnight growth. The cultures were diluted to the same OD_600_ of 1 and spotted at 10× serial dilutions on YPD plates containing 0.01% DMSO as control or 10 μg/ml CFW. 1 M D-sorbitol (MilliporeSigma) was used to provide external osmotic support. Plates were incubated at 30°C for at least 2.5 days before scanning.

### CHX chase assay

Degradation of endogenous Lsg1 protein after heat shock was evaluated as described previously (Ruan *et al*. 2017). Briefly, a log-phase culture of yeast expressing Lsg1-HA was heated at 42°C for 30 min with rotation. Recovery at 30°C was performed in the presence of 100 μg/ml CHX. At the indicated time points, the same volume of culture was collected, lysed by boiling and glass bead beating in LDS sample buffer, and subjected to immunoblotting analysis.

To assess the UPS-mediated protein degradation, Ub-R-EGFP expression was induced in the presence of 1 mM CuSO_4_ for 30 min at 30°C, followed by the treatment with 100 μg/ml CHX. At every 10-min interval, single-cell GFP fluorescence was measured by using Attune NxT flow cytometer equipped with appropriate filter sets. Background intensities prior to CuSO_4_ addition were subtracted from the mean GFP intensities at other time points. Relative intensities to the first time point (i.e. after 30 min CuSO_4_ treatment) were calculated and plotted.

### Hydropathy plots

Kyte–Doolittle hydropathy plots were generated using ProtScale (Kyte and Doolittle 1982; Gasteiger *et al*. 2005) with the following parameters: window size, 5 amino acids; relative weight of the window edges compared with the window center, 100%; linear weight variation model; no normalization. Transmembrane domain was predicted using TMHMM-2.0 (Möller *et al*. 2001).

### Statistical analysis

Descriptions of statistical tests and *P* values can be found in Figure Legends. Statistical analyses were performed with GraphPad Prism 6.0 and Microsoft Excel. No statistical methods were used to predetermine the sample size. The experiments were not randomized, and the investigators were not blinded to allocation during experiments and result assessment.

## Results

### *GAS1* is required for mitochondrial accumulation and degradation of misfolded proteins

**Fig. 1.**
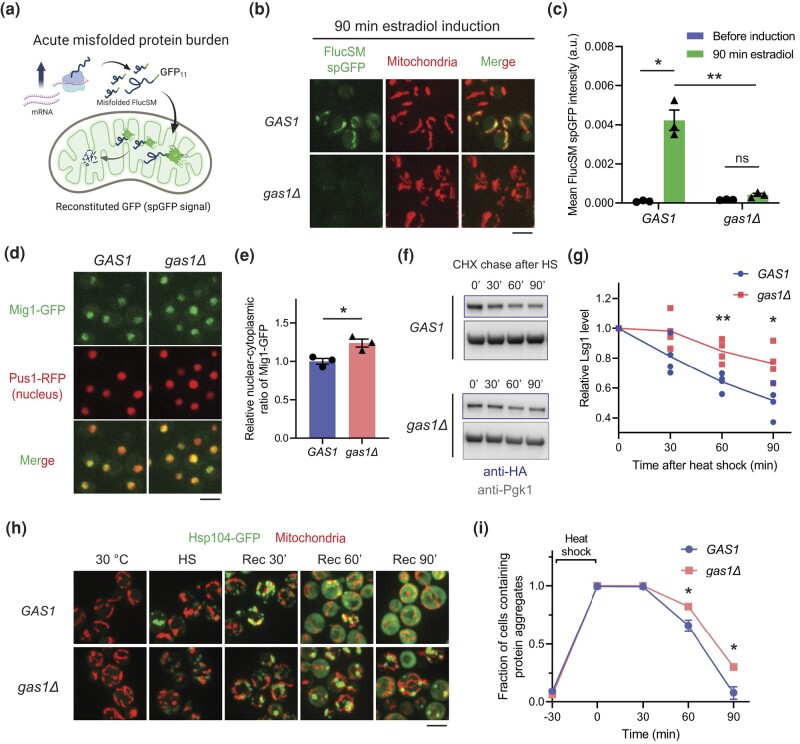
Deletion of *GAS1* inhibits mitochondrial accumulation and degradation of misfolded proteins. a) Schematic diagram of FlucSM split-GFP assay upon proteotoxic stress associated with elevated misfolded protein burden. b, c) Images b) and quantification c) of FlucSM spGFP signal in mitochondria of wild-type *GAS1* and *gas1Δ* cells after estradiol induction. Shown in c): means ± SEM of FlucSM spGFP intensities with an a.u. Paired 2-tailed *t*-test between no induction and estradiol treatment for 90 min. Unpaired 2-tailed *t*-test between *GAS1* and *gas1Δ* cells after 90 min induction. Three biological replicates. d, e) Images d) and quantification e) of the nuclear-cytoplasmic translocation of Mig1-GFP signal using Pus1-RFP as a nuclear marker. Retention of Mig1 in the nucleus indicates inactive Snf1 (yeast AMPK). Shown in e): means ± SEM of relative Mig-GFP nuclear-cytoplasmic ratios. Unpaired 2-tailed *t*-test. Three biological replicates. f, g) Immunoblots f) and quantifications g) showing data points and means of Lsg1-HA degradation in vivo after heat shock (HS) in the presence of CHX. Unpaired 2-tailed *t*-test. Four biological replicates. h, i) Formation and dissolution of protein aggregates labeled with Hsp104-GFP in *GAS1* and *gas1Δ* cells before and after heat shock at 42°C for 30 min. Rec, recovery at 30°C for 90 min. Shown in i): means ± SEM of fractions of cells containing protein aggregates. Unpaired 2-tailed *t*-test. Three biological replicates. For the last 2 time points where significant differences were observed, a total of 136 and 52 wild-type cells, and 165 and 79 *gas1Δ* cells were counted. *P < 0.05; **P < 0.01; ***P < 0.001; ns, not significant, *P* > 0.05. Scale bars, 5 μm.

We have recently conducted an unbiased genetic screen that uncovered potential MAGIC regulators in yeast by performing the split-GFP assay in the yeast non-essential knockout library (Wang *et al*. 2023). In brief, the endogenous Lsg1 protein and a destabilized firefly luciferase mutant FlucSM were tagged with the eleventh β-strand of GFP (GFP_11_), and the first 10 β-strands of GFP (GFP_1–10_) were targeted to mitochondrial matrix by fusing with the mitochondrial matrix protein Grx5 or the mitochondrial targeting sequence of Subunit 9 of mitochondrial ATPase (Su9 MTS) from *Neurospora crassa* (Gupta *et al*. 2011; Ruan *et al*. 2017; Wang *et al*. 2023). In wild-type cells, increased GFP fluorescence (spGFP signal) in mitochondria was observed under proteotoxic stresses such as heat shock (Ruan *et al*. 2017) or after acute overexpression of misfolded proteins under a β-estradiol–inducible system, by which the β-estradiol binds to a synthetic transcriptional factor and activates transcription under the *GAL1* promoter (Costa *et al*. 2018; Wang *et al*. 2023) (Fig. 1, a–c). We analyzed each mutant before and after the proteotoxic stress and identified gene deletions that gave rise to differential spGFP patterns compared with the wild-type cells (Wang *et al*. 2023).

*gas1Δ* was one of the mutants that failed to show an increased FlucSM spGFP signal after stress compared with the wild-type control (Fig. 1, b and c; Wang *et al*. 2023). Because activation of Snf1 kinase, the yeast homolog of human AMP-activated protein kinase (AMPK), inhibits the import of misfolded proteins into mitochondria (Wang *et al*. 2023), we first tested if Snf1 activity is elevated in *gas1Δ* by using the nuclear-cytoplasmic transport of Mig1-GFP as a reporter. Under glucose restriction, Mig1 is phosphorylated by active Snf1 and exported from the nucleus (De Vit *et al*. 1997; Wang *et al*. 2023). In *gas1Δ* cells, the Mig1-GFP signal remained in the nucleus and exhibited slightly more enrichment compared with the control (Fig. 1, d and e). This result argues against the possibility that the inhibition of MAGIC in *gas1Δ* cells is primarily caused by Snf1 activation. Moreover, we used immunoblots to assess the degradation of endogenous misfolded proteins and performed time-lapse imaging to track the disaggregation of cytosolic protein aggregates, both of which depend on mitochondrial import of misfolded proteins through MAGIC (Ruan *et al*. 2017). We found that the degradation of misfolded Lsg1 after heat shock was significantly delayed in *gas1Δ* (Fig. 1, f and g). The dissolution of protein aggregates labeled with protein disaggregase Hsp104 (Zhou *et al*. 2014; Ruan *et al*. 2017) was also impaired, even though the association of aggregates with mitochondria appeared unaffected (Fig. 1, h and i). In sum, these data suggest that *GAS1* is a regulator of the mitochondria-mediated pathway for degrading certain cytosolic aggregation-prone proteins.

### Re-analysis of the intracellular localization of Gas1-GFP reveals an unexpected mitochondrial targeting domain

**Fig. 2.**
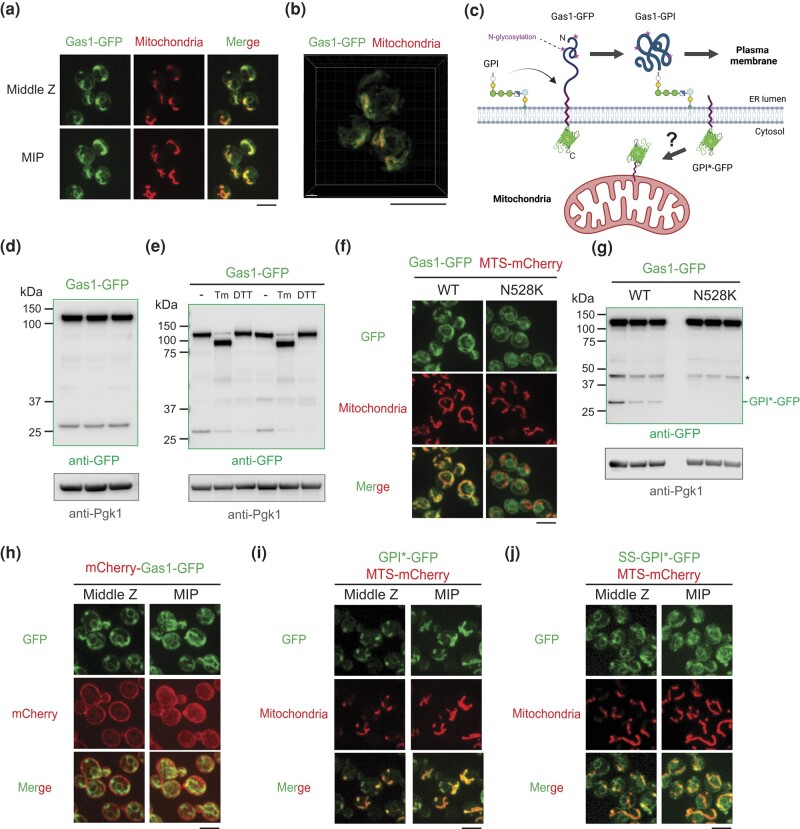
C-terminal GPI anchor signal of Gas1 protein localizes to mitochondria. a) Images of Gas1-GFP, showing partial colocalization with mitochondrial matrix labeled by MTS-mCherry. The ring-like structure may represent peri-nuclear ER and nuclear periphery as reported previously (Koch and Pillus 2009). MIP, maximal intensity projection. b) 3D rendering of super-resolution images of Gas1-GFP, showing GFP signals outside the mitochondrial matrix labeled with MTS-mCherry. c) Working model of GPI transfer and peptide cleavage during Gas1 maturation in the ER. d) Immunoblots of whole cell lysates from cells expressing Gas1-GFP. Three biological repeats. e) Immunoblots of whole cell lysates from cells expressing Gas1-GFP after tunicamycin (Tm) or DTT treatment. Two biological repeats. f, g) Images f) and immunoblots g) of whole cell lysates from cells expressing Gas1-GFP or Gas1-N528K-GFP. Asterisk, putative degradation products detected in all samples. Three biological repeats. h–j) Images of *gas1Δ* cells expressing mCherry-Gas1-GFP h), and wild-type cells expressing GPI*-GFP i) or SS-GPI*-GFP j). MIP, maximal intensity projection. Scale bars, 5 μm.

It was previously reported that Gas1 protein localizes to mitochondria in addition to the cell surface, ER, and nuclear periphery (Huh *et al*. 2003; Koch and Pillus 2009). It was therefore reasonable for us to hypothesize that the mitochondrial localization of Gas1 is related to its role in MAGIC. To test this, it was necessary to gain an understanding of how this purported cell wall protein is targeted to mitochondria. By using confocal microscopy to observe the subcellular localization of the C-terminally tagged Gas1-GFP, we confirmed that a portion of GFP fluorescence colocalized with mitochondria labeled with MTS-mCherry, and the other fraction of GFP fluorescence appeared to be in the ER and nuclear periphery (Fig. 2a), which was reported previously (Koch and Pillus 2009). By using super-resolution microscopy, we found that mitochondrial Gas1-GFP signal delineated the contour of the mitochondrial matrix, suggesting its localization in the mitochondrial membranes or intermembrane space (Fig. 2b). Importantly, our Gas1-GFP strain was resistant to treatment with CFW, a cell wall destabilizing reagent (Ram and Klis 2006), whereas *gas1Δ* mutant failed to grow on CFW-containing plates (Supplementary Fig. 1a). These data suggest that C-terminal GFP tagging of Gas1 preserves its function in maintaining cell wall integrity.

The trafficking and post-translational modification of Gas1 are 2 intricately coupled processes (Nuoffer *et al*. 1991, 1993; Doering and Schekman 1996; Popolo and Vai 1999; Ragni *et al*. 2007). To reach the cell surface, newly synthesized Gas1 protein is targeted to ER by a canonical N-terminal signal sequence (SS) (Nuoffer *et al*. 1991; Popolo and Vai 1999; Ragni *et al*. 2007). A cleavable hydrophobic sequence at the C-terminus temporarily anchors Gas1 on the ER membrane and serves as a GPI anchor signal (GPI*) that is necessary for the addition of a GPI moiety via the GPI anchor transamidase complex (Nuoffer *et al*. 1991; Doering and Schekman 1996; Ragni *et al*. 2007). As a result, we conceived that the cleaved GPI* should bear the GFP tag (GPI*-GFP), and the GPI-anchored Gas1 (Gas1-GPI) is transported to plasma membrane through the secretory pathway and progressively modified by N-glycosylation and O-glycosylation in the ER and Golgi (Nuoffer *et al*. 1991; Doering and Schekman 1996; Fig. 2c). In line with this model, anti-GFP immunoblot analysis on the yeast whole cell lysate of Gas1-GFP strain showed 2 notable species (Fig. 2d). A high molecular weight (MW) species exhibited the expected size of N-glycosylated and uncleaved Gas1-GFP (∼120 kDa), as confirmed by treatment with tunicamycin (Tm), an inhibitor of N-acetylglucosamine transferases (Kuo and Lampen 1974), which decreased its MW to that of unmodified Gas1-GFP (∼90 kDa; (Fig. 2, d and e). By contrast, treatment with DTT, which reduces disulfide bonds of ER proteins and elicits the unfolded protein stress (Kimata and Kohno 2011), had no effect on N-glycosylation (Fig. 2e). The low MW species appeared to be comparable to GPI*-GFP (∼28 kDa; Fig. 2d). Interestingly, either treatment with tunicamycin or DTT reduced the amount of low MW species, suggesting that ER stress may compromise the cleavage of GPI* regardless of the presence of N-glycosylation (Fig. 2e).

We next tested if the cleavage of GPI* is responsible for the mitochondrial localization of Gas1. The amino acid sequence at and adjacent to the GPI anchor attachment site of Gas1 (N528) is important for efficient peptide cleavage (Nuoffer *et al*. 1993). Mutation of N528 to a lysine residue (N528K) abolishes the glycolipid transfer and peptide cleavage, and as a result, Gas1 remains unprocessed and only N-glycosylated in the ER (Nuoffer *et al*. 1993). We found that Gas1-N528K-GFP protein only localized to ER and failed to exhibit any mitochondrial localization (Fig. 2f) and as expected the low MW species also disappeared in the anti-GFP immunoblot (Fig. 2g). This result indicates that mitochondrial GFP fluorescence requires the cleavage of GPI*.

To differentially observe the N- and C-terminal portion of the Gas1 protein after GPI* cleavage, we fused the Gas1 protein with a mCherry at its N terminus after the ER-targeting signal peptide, and simultaneously with a GFP at its C-terminus following the GPI anchor signal to form the mCherry-Gas1-GFP protein. The fusion protein was able to support growth in both DMSO control and CFW-containing medium, suggesting that the cell wall function was preserved (Supplementary Fig. 1b). The mCherry signal was mostly detected on the cell periphery where the GFP fluorescence was largely absent, whereas only GFP but not mCherry signal was detected on mitochondria (Fig. 2h). Only at the ER/nuclear periphery were both mCherry and GFP colocalized, which likely represented the unprocessed mCherry-Gas1-GFP (Fig. 2h). This dual-color pattern further confirms that after GPI* cleavage, the N-terminal main portion of the protein is trafficked to the cell periphery whereas GPI* is targeted to mitochondria.

To determine if GPI* is sufficient for mitochondrial localization, we ectopically expressed the C-terminally tagged GPI*-GFP or with an additional N-terminal ER-targeting signal sequence (SS-GPI*-GFP) under the *GAS1* promoter. GPI*-GFP signal was predominantly localized to mitochondria, whereas SS-GPI*-GFP showed both mitochondrial and more predominantly, ER localization (Fig. 2, i and j). These data suggest that the GPI anchor signal of Gas1 could be a mitochondrial targeting sequence even in the presence of an ER-targeting sequence.

### Enzymatic activity of Gas1 rather than its GPI anchor signal is required for MAGIC

**Fig. 3.**
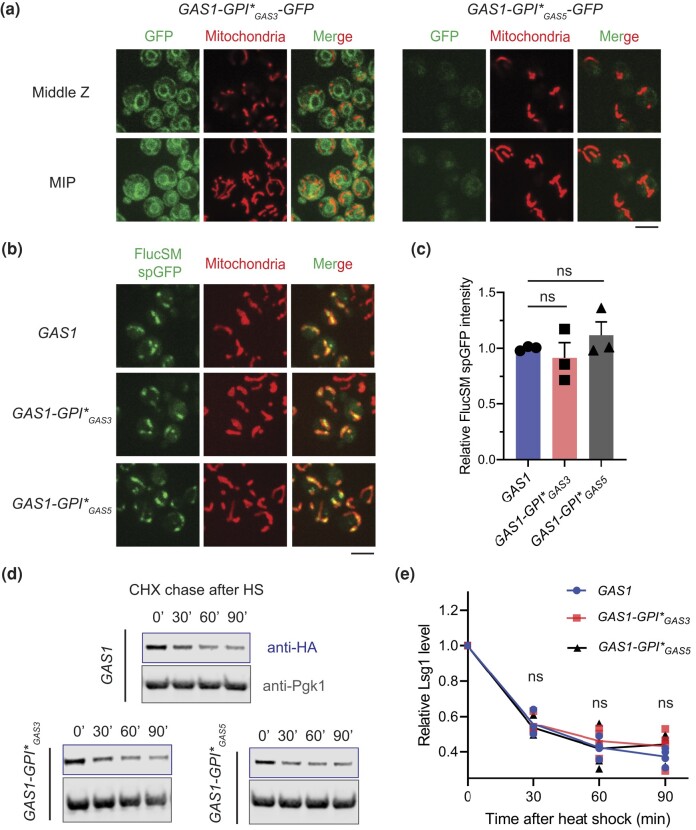
Mitochondria-associated GPI anchor signal of Gas1 protein is not required for MAGIC. a) Images of GAS1-GPI*_GAS3_-GFP or GAS1-GPI*_GAS5_-GFP cells showing minimal colocalization with mitochondria labeled with MTS-mCherry. MIP, maximal intensity projection. b, c) Images b) and quantification c) of FlucSM spGFP signal in mitochondria of GAS1-GPI*_GAS3_ or GAS1-GPI*_GAS5_ cells. Shown in c): means ± SEM of relative FlucSM spGFP intensities. Unpaired 2-tailed *t*-test. Three biological replicates. d, e) Immunoblots d) and quantifications e) of Lsg1-HA degradation in vivo after heat shock (HS) in the presence of CHX. No significant differences were observed across all time points. Unpaired 2-tailed *t*-test. Four biological replicates. ns, not significant, *P* > 0.05. Scale bars, 5 μm.

Next, we asked if the mitochondria-associated GPI anchor signal of Gas1 is required for maintaining MAGIC, a process dependent on functional mitochondrial import (Ruan *et al*. 2017). To avoid affecting the targeting to plasma membrane and function of Gas1 in the cell wall, we replaced the endogenous GPI anchor signal of Gas1 by that of Gas3 and Gas5, 2 closely related Gas family glucanosyltransferases with C-terminal GPI anchor signals (Supplementary Fig. 1c; Ragni *et al*. 2007). Yeast strains expressing recombinant proteins (GAS1-GPI*_GAS3_ and GAS1-GPI*_GAS5_ strains) were resistant to treatment with CFW, and these proteins were fully glycosylated at a level comparable to the wild-type Gas1 (Supplementary Fig. 1, d and e), indicating normal processing and cell wall function of the recombinant Gas1 proteins. Importantly, by C-terminal tagging with GFP, neither recombinant protein exhibited mitochondrial localization: the fluorescence was confined to the ER in GAS1-GPI*_GAS3_-*GFP* strain, whereas the overall signal was modest in GAS1-GPI*_GAS5_-*GFP* strain (Fig. 3a). We then asked if MAGIC is preserved in the absence of the mitochondria-associated GPI*. Mitochondrial accumulation of misfolded FlucSM after acute overexpression was not affected by GPI* replacement (Fig. 3, b and c). Misfolded Lsg1 was also degraded normally after heat shock (Fig. 3, d and e). We therefore concluded that the GPI anchor signal of Gas1 and its mitochondrial localization are not involved in the regulation of MAGIC.

**Fig. 4.**
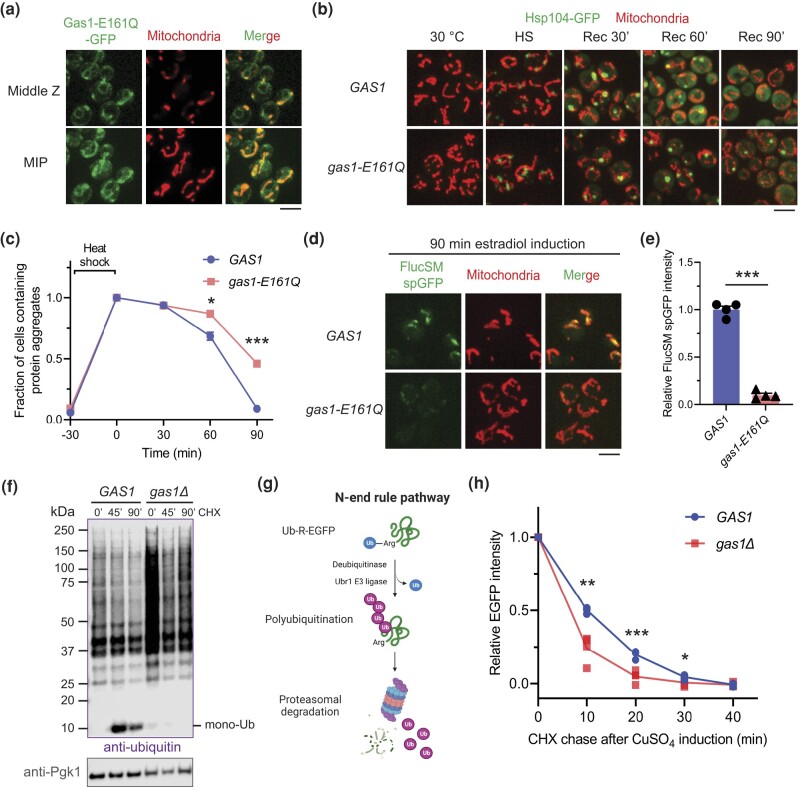
Gas1 regulates both MAGIC and UPS-mediated protein degradation. a) Images of cells expressing an ectopic copy of Gas1-E161Q-GFP and MTS-mCherry. MIP, maximal intensity projection. b, c) Formation and dissolution of protein aggregates labeled with Hsp104-GFP in wild-type *GAS1* and *gas1-E161Q* cells before and after heat shock (HS) at 42°C for 30 min. Rec, recovery at 30°C for 90 min. Shown in c): means ± SEM of fractions of cells containing protein aggregates. Unpaired 2-tailed *t*-test. Three biological replicates. For the last 2 time points where significant differences were observed, a total of 317 and 338 wild-type cells, and 308 and 364 *gas1-E161Q* cells were counted. d, e) Images d) and quantification e) of FlucSM spGFP in mitochondria of *GAS1* and *gas1-E161Q* cells after estradiol treatment for 90 min. Shown in e): means ± SEM of relative FlucSM spGFP intensities. Unpaired 2-tailed *t*-test. Four biological replicates. f) Anti-ubiquitin immunoblots of whole cell lysates, showing an accumulation of total protein ubiquitination at high MW in *gas1Δ* cells. Mono-Ub: monomer ubiquitin. g) Diagram of N-end rule pathway using Ub-R-EGFP as a UPS substrate. h) Degradation of copper (CuSO_4_)-inducible Ub-R-EGFP in vivo in wild-type and *gas1Δ* cells. Means of single-cell GFP intensities were measured every 10 min during CHX chase for 40 min. Unpaired 2-tailed *t*-test. Four biological repeats. *P < 0.05; **P < 0.01; ***P < 0.001. Scale bars, 5 μm.

We next tested if the glucanosyltransferase activity of Gas1 is required for MAGIC by studying the effect of the catalytically inactive *gas1-E161Q* mutation (Carotti *et al*. 2004; Koch and Pillus 2009), which also caused CFW sensitivity (Supplementary Fig. 1f). Like the wild-type protein, Gas1-E161Q was fully glycosylated and likely trafficked to the cell surface (Supplementary Fig. 1g). Gas1-E161Q-GFP also showed both ER and mitochondrial GFP localization, indicative of normal cleavage of GPI* (Fig. 4a). But like *gas1Δ*, this mutant exhibited defects in proteostasis and MAGIC, including delayed dissolution of protein aggregates after heat shock (Fig. 4, b and c) and reduced mitochondrial import of misfolded FlucSM (Fig. 4, d and e). These results suggest that the enzymatic activity of Gas1 is essential for MAGIC.

To determine whether loss of cell wall integrity could inhibit MAGIC in general, we examined 2 other cell wall mutants with the deletion of *BGL2* encoding an endo-β-1,3-glucanase (Mrsa *et al*. 1993), or deletion of *GAS5* encoding another β-1,3-glucanosyltransferase (Popolo and Vai 1999; Ragni *et al*. 2007). Both mutants showed milder sensitivity to CFW than *gas1Δ* (Supplementary Fig. 2a) and a moderate reduction in mitochondrial import of FlucSM (Supplementary Fig. 2b), which was less pronounced than in *GAS1*-deficient cells (*gas1Δ*: Fig. 1c; *gas1-E161Q*: Fig. 4e). This analysis indicates that cell wall maintenance is indeed important for MAGIC. Because loss of *GAS1* could create a hypo-osmotic-like stress (Ram *et al*. 1998; Turchini *et al*. 2000), we asked if providing external osmotic support to *gas1Δ* cells can rescue the defect in MAGIC. It was reported that *gas1Δ* cells in the osmotically stabilized medium with 1 M sorbitol became resistant to SDS, a destabilizing reagent for the plasma membrane (Turchini *et al*. 2000). We found that *gas1Δ* cells also regained partial resistance to CFW in the presence of 1 M sorbitol (Supplementary Fig. 2c). However, no effect on the mitochondrial import of misfolded FlucSM was observed in both wild-type and *gas1Δ* cells (Supplementary Fig. 2d). Therefore, partial rescue of the cell wall defect may not be sufficient to relieve the inhibition of MAGIC.

### Effect of *GAS1* deficiency on UPS

To gain insights into whether *GAS1* plays a broader role in other proteostasis pathways, we examined the effect of *gas1Δ* on UPS. Surprisingly, anti-ubiquitin immunoblot analysis on the whole cell lysate revealed a drastic increase in total protein ubiquitination compared with the wild-type cell (Fig. 4f). After inhibiting protein synthesis by CHX, ubiquitinated proteins were gradually degraded, but deconjugated mono-ubiquitin was barely detected in *gas1Δ* cells (Fig. 4f). To understand if the increased total protein ubiquitination was due to activation of conjugation reactions or inhibition of proteasomal degradation, we evaluated the degradation kinetics of the Ub-R-EGFP, a non-misfolded N-end rule substrate for UPS (Fig. 4g; Bachmair *et al*. 1986; Heessen *et al*. 2003; Samant *et al*. 2018)]. In contrast to the delayed degradation of MAGIC substrate, loss of *GAS1* resulted in significantly faster degradation of Ub-R-EGFP (Fig. 4h), suggesting that ubiquitination reactions may be more active in the mutant to shunt more proteins to proteasomal degradation. In summary, our data demonstrates that loss of Gas1 activity elevates UPS-mediated degradation but inhibits MAGIC.

## Discussion

Gas1 in the budding yeast is a GPI-anchored β-1,3-glucanosyltransferase that elongates the β-1,3-glucan chains and plays a critical role in the dynamic remodeling of the cell wall (Popolo and Vai 1999; Ragni *et al*. 2007). *GAS1*-deficient cells show cell wall defects characterized by swollen cell volume, round shape, CFW sensitivity, reduced growth rate, and decreased cell viability during the stationary phase (Popolo *et al*. 1993; Ram *et al*. 1998; Levin 2005; Ram and Klis 2006). *GAS1* deficiency also triggers the cell wall integrity signaling pathway including the mitogen-activated protein (MAP) kinase cascade and elicits compensatory responses such as an increase in chitin synthesis and deposition to rescue cell wall integrity (Ram *et al*. 1998; Turchini *et al*. 2000; Levin 2005). In addition to the established function in the cell wall, it was reported that *GAS1* may play roles in several intracellular processes such as DNA damage response (Eustice and Pillus 2014), locus-specific transcriptional silencing (Koch and Pillus 2009), and ER stress response (Cui *et al*. 2019). Deletion of *GAS1* increases cellular sensitivity to DNA damages caused by genotoxins (Eustice and Pillus 2014), and loss of its enzymatic activity leads to defective telomeric silencing and elevated rDNA silencing (Koch and Pillus 2009). Because C-terminally tagged Gas1-GFP exhibits intracellular localizations in ER, nuclear periphery, and mitochondria (Huh *et al*. 2003; Koch and Pillus 2009), it has been proposed that Gas1 is involved in post-translational glycosylation of putative chromatin components (Koch and Pillus 2009; Eustice and Pillus 2014). Loss of *GAS1* also elevates the unfolded protein response (UPR) and renders the mutant cells resistant to tunicamycin-induced ER proteotoxic stress (Cui *et al*. 2019). Both UPR and ER-associated degradation maintain proteostasis in the ER and may in turn promote the secretion and glycosylation of essential plasma membrane and cell wall proteins (Scrimale *et al*. 2009). However, detailed mechanisms underlying the intracellular functions of Gas1 remain unclear.

Mitochondria are important organelles in cellular metabolism and cytosolic proteostasis (Wai and Langer 2016; Ruan *et al*. 2017, 2020; Andréasson *et al*. 2019; Wang *et al*. 2023). Loss of cytosolic proteostasis is manifested by protein misfolding and formation of protein aggregates, which are often tethered to intracellular organelles such as ER and mitochondria (Escusa-Toret *et al*. 2013; Zhou *et al*. 2014). Under proteotoxic stress, certain misfolded cytosolic proteins can be translocated into and degraded inside mitochondria through MAGIC pathway (Ruan *et al*. 2017; Wang *et al*. 2023). We have recently conducted a genetic screening in yeast to uncover potential regulators of MAGIC (Wang *et al*. 2023). In the present study, we elected to focus on one of the hits, Gas1, due to its interesting mitochondrial localization. Our data suggest that Gas1 is involved in cytosolic proteostasis mechanisms including MAGIC and UPS. Specifically, *GAS1* deficiency inhibits the mitochondrial accumulation of FlucSM and degradation of aggregation-prone Lsg1 but promotes UPS-mediated degradation of a non-misfolded N-end rule substrate. In the wild-type cells misfolded FlucSM or Lsg1 is primarily degraded through MAGIC by the mitochondrial Lon protease (Ruan *et al*. 2017; Wang *et al*. 2023). How loss of *GAS1* disrupts this process remains an open question. Future studies may focus on testing if mitochondrial import or Lon-mediated degradation is suppressed in this mutant. The other caveat of this study is that most of our observations were based on a few model substrates such as FlucSM, Lsg1, and Ub-R-EGFP. So, the generality of our observations remains to be substantiated.

While investigating if mitochondrial targeting is important for the role of *GAS1* in MAGIC, we unexpectedly identified the C-terminal GPI anchor signal of Gas1 (GPI*_Gas1_) as an unconventional mitochondrial targeting sequence. GPI*_Gas1_ does not bear a typical amphiphilic helix like Su9 MTS from *N. crassa* but likely contains a hydrophobic transmembrane domain, which was not present in GPI*_Gas3_ or GPI*_Gas5_ (Supplementary Fig. 3). It is worth noting that a previous study showed that GPI*_Gas1_ with an N-terminal GFP tag (denoted as GFP-Gas1-GPI) only exhibits ER localization (Ast *et al*. 2013). We speculate that a well-folded GFP at the N terminus may hinder the import or association of GPI* with mitochondria. GPI* may have a dual targeting specificity that allows its route to ER when mitochondrial targeting is impaired. Anti-GPI*_Gas1_ immunoblots on mitochondrial fraction or immunofluorescence staining may be necessary to further validate its subcellular localization. Also, it will be interesting to test if some other GPI-anchored proteins in yeast or higher organisms contain a GPI anchor signal with similar hydrophobicity and mitochondrial targeting capability.

By replacing the GPI* of Gas1 with that of Gas3 or Gas5, we showed that this mitochondria-associated peptide is dispensable for mitochondrial accumulation of misfolded FlucSM and post-heat shock degradation of misfolded Lsg1. By contrast, the catalytically inactive Gas1-E161Q protein exhibited normal maturation and mitochondrial localization, but still failed to maintain MAGIC. Importantly, 2 other cell wall mutants with moderate CFW sensitivity also displayed mild inhibition of MAGIC, suggesting that cell wall integrity may be important for MAGIC. The stronger phenotypes of *gas1Δ* or *gas1-E161Q* mutant than others in terms of CFW sensitivity and MAGIC inhibition may be attributed to either more severe cell wall defects or specific effects that remain to be determined. At least the partial rescue of the cell wall defect in *gas1Δ* by osmotic stabilizer failed to restore MAGIC. Although it was reported that the MAP kinase signaling pathway that senses the cell wall stress is required for mitophagy during nitrogen starvation (Mao *et al*. 2011), whether this pathway regulates MAGIC and UPS in the nutrient-rich medium used in this study (in which mitophagy and autophagy are typically not active) is unknown. MAGIC is inhibited during caloric restriction primarily due to activation of Snf1 (yeast AMPK)-dependent transcriptional reprogramming, but not elevated autophagy (Wang *et al*. 2023). In this study, we showed that AMPK remains inactive in *gas1Δ* cells, thus unlikely contributing to the MAGIC defect.

Interestingly, certain yeast cell wall proteins and RNA-binding proteins were shown to have amyloid-forming properties in vivo or in vitro (Kalebina *et al*. 2008; Ryzhova *et al*. 2018; Sergeeva *et al*. 2019). As the present and previous work demonstrated that some amyloid-forming proteins (Gas1, Gas5, Bgl2, and Nsr1) are involved in the regulation of MAGIC (Wang *et al*. 2023), it would be interesting to examine if other amyloid proteins have similar physiological functions in MAGIC or other proteostasis pathways. Although the C-terminally truncated Gas1 lacking the serine-rich domain and GPI anchor signal is able to form amyloid-like intracellular aggregates (Ryzhova *et al*. 2018), we did not observe aggregation properties of the wild-type or mutant Gas1 protein with a fluorescence tag. However, it is still possible that the amyloid property of Gas1 facilitates the formation of microdomains in the plasma membrane at the mother-bud neck and bud scars (Rolli *et al*. 2009). Our results also cannot rule out the possibility that the intracellular fraction of Gas1 in the ER and nuclear periphery, albeit constituting a minor fraction of total Gas1, plays a direct role in proteostasis.

## Supplementary Material

jkad263_Supplementary_Data

## Data Availability

The authors affirm that all data necessary for confirming the conclusions are present within the article, figures, and tables. Requests for strains and plasmids should be directed to and will be fulfilled by the corresponding author, RL (rong@jhu.edu). Supplemental material available at G3 online.
