# Supplementary material for: GPI-anchored Gas1 protein regulates cytosolic proteostasis in budding yeast
Source: G3 (Bethesda). 2024 Jan 30;14(3):jkad263. doi: 10.1093/g3journal/jkad263 (PMC10917523; doi:10.1093/g3journal/jkad263)
Supplement: jkad263_Supplementary_Data [file jkad263_supplementary_data.zip › Supplemental_Figure_Legends_G3-2023-404628.docx]

**Legends for Supplemental Figures**

**Figure S1. Characterization of *GAS1*-related mutants.** (**A, B, D, F**) Yeast spotting assays on YPD plates containing DMSO or calcofluor white (CFW). Ten-fold serial dilutions from left to right for each plate. (**C**) C-terminal sequences of Gas1, Gas3, and Gas5 protein. Red, key basic residues; Bold, GPI attachment sites; Green, GPI anchor signals to be removed in matured proteins. (**E, G**) Anti-Gas1 immunoblots of whole cell lysates, showing that N- and O-glycosylation through ER and Golgi is normal for both GPI*-replaced variants and catalytically inactive Gas1-E161Q protein.

**Figure S2. Loss of cell wall integrity may contribute to the inhibition of MAGIC, which is not rescued by external osmotic support. (A)** Yeast spotting assays on YPD plates containing DMSO or calcofluor white (CFW). (**B**) Quantification of mitochondrial FlucSM spGFP signal in wild-type cells, *bgl2Δ* and *gas5Δ* mutants. Means ± SEM of relative FlucSM spGFP intensities are shown. Unpaired two-tailed *t*-test. Three biological replicates. ***P* < 0.01. (**C)** Growth of *GAS1*, *GAS1-GFP*, and *gas1Δ* cells in YPD plates containing DSMO, CFW, or CFW plus 1 M sorbitol as external osmotic support. (**D**) Quantification of FlucSM spGFP signal in mitochondria in *GAS1* or *gas1**Δ* cells growing in SC medium plus 1M sorbitol. Paired two-tailed *t*-test. Three biological replicates. ns, not significant.

**Figure S3. Kyte-Doolittle hydropathy plots for Su9 MTS and GPI anchor signals.** Su9 MTS is the N-terminal 69 residues of ATP synthase subunit 9 from *Neurospora crassa* (primary accession: P00842). GPI* of yeast Gas1, Gas3, or Gas5 protein represents the corresponding GPI anchor signal peptide (see Figure S1C). Kyte-Doolittle hydropathy plots were generated by ProtScale. TMHMM-2.0 was used to predict the hydrophobic transmembrane domain (highlighted in red).
