# Supplementary material for: GPI-anchored Gas1 protein regulates cytosolic proteostasis in budding yeast
Source: G3 (Bethesda). 2024 Jan 30;14(3):jkad263. doi: 10.1093/g3journal/jkad263 (PMC10917523; doi:10.1093/g3journal/jkad263)
Supplement: jkad263_Supplementary_Data [file jkad263_supplementary_data.zip › Table_S1_G3-2023-404628.docx]

**Table S1. List of yeast strains and plasmids**

| **Yeast** | **Genotype** | **Source** |
| --- | --- | --- |
| BY4741 | *MATa his3Δ1; leu2Δ0; met15Δ0; ura3Δ0* | Parental strain |
| RLY9803 | *TRP1::P_GPD_-GRX5-HA-GFP_1-10_::NatMX6; AMP::GEM-P_GAL1_-FlucSM-HA-GFP_11_::KanMX6; TOM70-mCherry::Ura3MX6* | Wang et al. 2023 |
| RLY9806 | *MIG1-GFP::His3MX6; PUS1-RFP::HphMX6* | Wang et al. 2023 |
| RLY9860 | *TRP1::P_GPD_-GRX5-HA-GFP_1-10_::NatMX6; AMP::GEM-P_GAL1_-FlucSM-HA-GFP_11_::KanMX6; TOM70-mCherry::Ura3MX6; gas1Δ::His3MX6* | This study |
| RLY9861 | *MIG1-GFP::His3MX6; PUS1-RFP::HphMX6; gas1Δ::Leu2* | This study |
| RLY9862 | *TRP1::P_GPD_-MTS-mCherry-GFP_1-10_::NatMX6; LSG1-HA-GFP_11_::HisMX6* | This study |
| RLY9863 | *TRP1::P_GPD_-MTS-mCherry-GFP_1-10_::NatMX6; LSG1-HA-GFP_11_::HisMX6; gas1Δ::KanMX6* | This study |
| RLY9864 | *HSP104-GFP::His3MX6; TRP1::P_GPD_-MTS-mCherry::NatMX6* | Ruan et al. 2017 |
| RLY9865 | *HSP104-GFP::His3MX6; TRP1::P_GPD_-MTS-mCherry::NatMX6; gas1 Δ::KanMX6* | This study |
| RLY9866 | *GAS1-GFP::His3MX6* | Huh et al. 2003 |
| RLY9867 | *GAS1-GFP::His3MX6; TRP1::P_GPD_-MTS-mCherry::NatMX6* | This study |
| RLY9868 | *gas1Δ::KanMX6* | This study |
| RLY9869 | *ho::P_GAS1_-gas1-N528K-GFP::HphMX6; TRP1::P_GPD_-MTS-mCherry::NatMX6* | This study |
| RLY9870 | *gas1Δ::KanMX6; ho::P_GAS1_-mCherry-GAS1-GFP::His3MX6::HphMX6* | This study |
| RLY9871 | *ho::P_GAS1_-GPI*-GFP::HphMX6; TRP1::P_GPD_-MTS-mCherry::NatMX6* | This study |
| RLY9872 | *ho::P_GAS1_-SS-GPI*-GFP::HphMX6; TRP1::P_GPD_-MTS-mCherry::NatMX6* | This study |
| RLY9873 | *GAS1-GPI*_GAS3_::KanMX6* | This study |
| RLY9874 | *GAS1-GPI*_GAS5_::KanMX6* | This study |
| RLY9875 | *GAS1-GPI*_GAS3_-GFP::His3MX6::KanMX6; TRP1::P_GPD_-MTS-mCherry::NatMX6* | This study |
| RLY9876 | *GAS1-GPI*_GAS5_-GFP::His3MX6::KanMX6; TRP1::P_GPD_-MTS-mCherry::NatMX6* | This study |
| RLY9877 | *TRP1::P_GPD_-MTS-mCherry-GFP_1-10_::NatMX6; LSG1-HA-GFP_11_::HisMX6; GAS1-GPI*_GAS3_::KanMX6* | This study |
| RLY9878 | *TRP1::P_GPD_-MTS-mCherry-GFP_1-10_::NatMX6; LSG1-HA-GFP_11_::HisMX6; GAS1-GPI*_GAS5_::KanMX6* | This study |
| RLY9879 | *HSP104-GFP::His3MX6; TRP1::P_GPD_-MTS-mCherry::NatMX6; gas1Δ::KanMX6::Leu2::P_GAS1_-gas1-E161Q* | This study |
| RLY9880 | *ho::P_GAS1_- gas1-E161Q-GFP::HphMX6; TRP1::P_GPD_-MTS-mCherry::NatMX6* | This study |
| RLY9881 | *TRP1::P_GPD_-GRX5-HA-GFP_1-10_::NatMX6; AMP::GEM-P_GAL1_-FlucSM-HA-GFP_11_::KanMX6; TOM70-mCherry::Ura3MX6; gas1Δ::His3MX6:: KanMX6::Leu2-P_GAS1_-gas1-E161Q* | This study |
| RLY9882 | *TRP1::P_CUP1_-Ub-R-EGFP::NatMX6* | This study |
| RLY9883 | *TRP1::P_CUP1_-Ub-R-EGFP::NatMX6; gas1Δ::KanMX6* | This study |
| RLY9884 | *bgl2Δ::KanMX6* | Giaever et al. 2002 |
| RLY9885 | *gas5Δ::KanMX6* | Giaever et al. 2002 |
| RLY9886 | *ura3Δ0::GEM::HphMX6; TRP1::P_GPD_-GRX5-HA-GFP_1-10_::NatMX6; ho::P_GAL1_-FlucSM-HA-GFP_11_::His3MX6; TOM70-mCherry::Ura3MX6* | Wang et al. 2023 |
| RLY9887 | *ura3Δ0::GEM::HphMX6; trp1::P_GPD_-GRX5-HA-GFP_1-10_::NatMX6; HO::P_GAL1_-FlucSM-HA-GFP_11_::His3MX6; TOM70-mCherry::Ura3MX6; bgl2Δ::KanMX6* | This study |
| RLY9888 | *ura3Δ0::GEM::HphMX6; trp1::P_GPD_-GRX5-HA-GFP_1-10_::NatMX6; HO::P_GAL1_-FlucSM-HA-GFP_11_::His3MX6; TOM70-mCherry::Ura3MX6; gas5Δ::KanMX6* | This study |
| RLY9889 | *ura3Δ0::GEM-HphMX6; TRP1::P_GPD_-GRX5-HA-GFP_1-10_::NatMX6; ho::P_GAL1_-FlucSM-HA-GFP_11_::His3MX6; TOM70-mCherry::Ura3MX6; GAS1-GPI*_GAS3_::KanMX6* | This study |
| RLY9890 | *ura3Δ0::GEM-HphMX6; TRP1::P_GPD_-GRX5-HA-GFP_1-10_::NatMX6; ho::P_GAL1_-FlucSM-HA-GFP_11_::His3MX6; TOM70-mCherry::Ura3MX6; GAS1-GPI*_GAS5_::KanMX6* | This study |
|  |  |  |
| **Plasmid** | **Construct** | **Source** |
| RLB918 | *TRP1::P_GPD_-MTS-mCherry::NatMX6* | Ruan et al. 2017 |
| RLB1051 | *GEM-P_GAL1_-FlucSM-HA-GFP_11_::KanMX6* | Wang et al. 2023 |
| RLB1075 | *pRS316-ho(homology)-P_GAS1_-gas1-N528K-GFP::HphMX6-ho(homology)* | This study |
| RLB1076 | *pRS316-ho(homology)-P_GAS1_-mCherry-GAS1::HphMX6-ho(homology)* | This study |
| RLB1077 | *pRS316-ho(homology)-P_GAS1_-GPI*-GFP::HphMX6-ho(homology)* | This study |
| RLB1078 | *pRS316-ho(homology)-P_GAS1_-SS-GPI*-GFP::HphMX6-ho(homology)* | This study |
| RLB1079 | *P_GAS1_-gas1-E161Q::Leu2::KanMX6* | This study |
| RLB1080 | *TRP1::P_CUP1_-Ub-R-EGFP::NatMX6* | This study |
| RLB1081 | *pRS316-ho(homology)-P_GAS1_-gas1-E161Q-GFP::HphMX6-ho(homology)* | This study |
