# Supplementary figures and images for: GPI-anchored Gas1 protein regulates cytosolic proteostasis in budding yeast
Source: G3 (Bethesda). 2024 Jan 30;14(3):jkad263. doi: 10.1093/g3journal/jkad263 (PMC10917523; doi:10.1093/g3journal/jkad263)

Figure S3

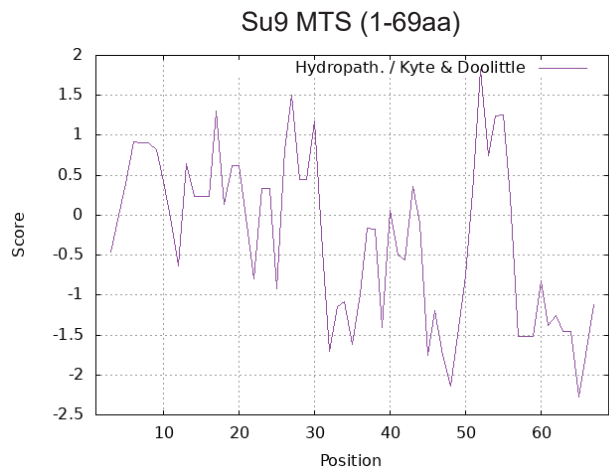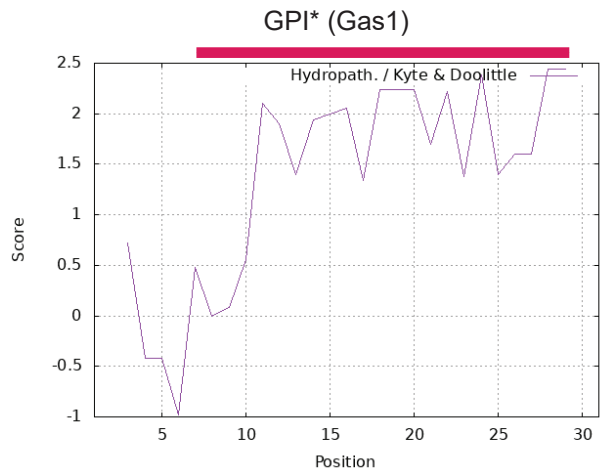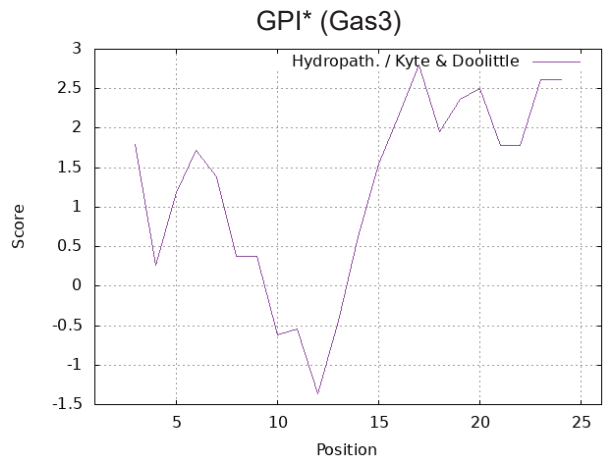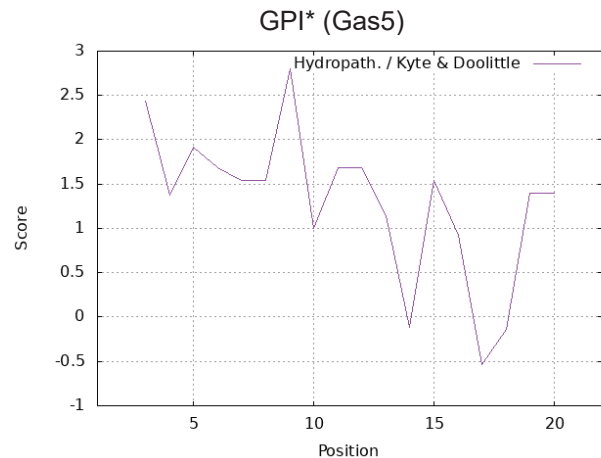

Supplement: jkad263_Supplementary_Data [file jkad263_supplementary_data.zip › Figure_S3_G3-2023-404628.pdf]

Figure S1

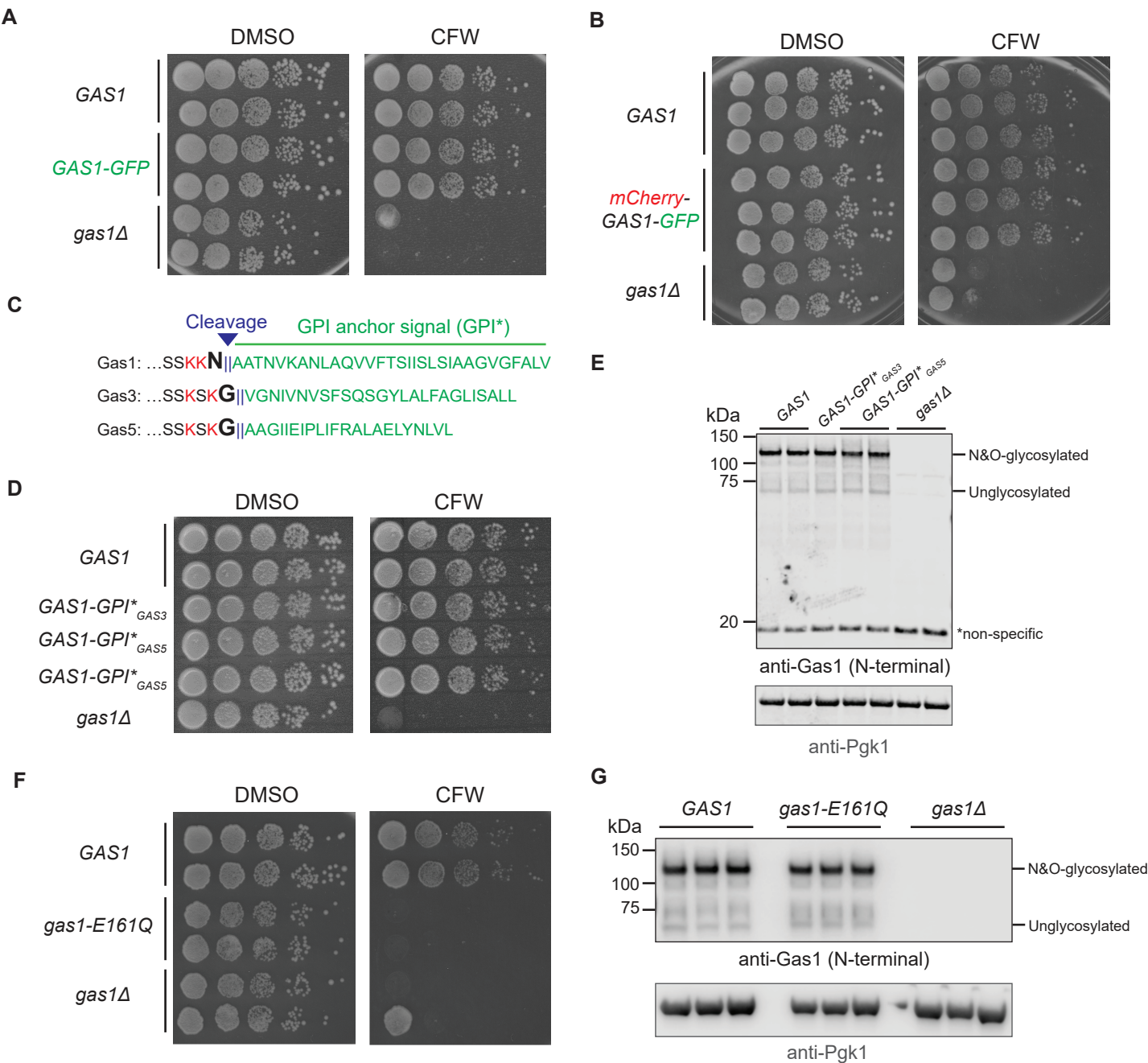

Supplement: jkad263_Supplementary_Data [file jkad263_supplementary_data.zip › Figure_S1_G3-2023-404628.pdf]

**Figure S2**

**A**

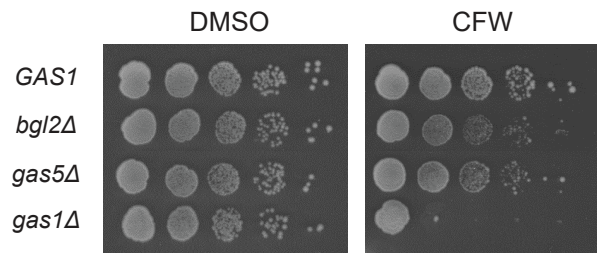

**B**

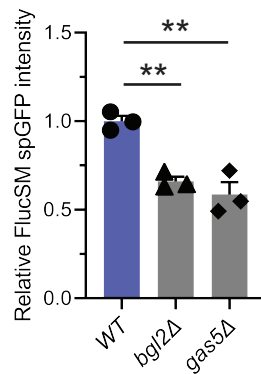

**C**

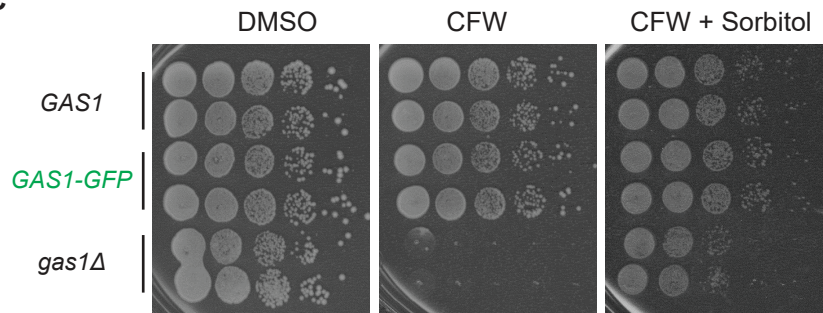

**D**

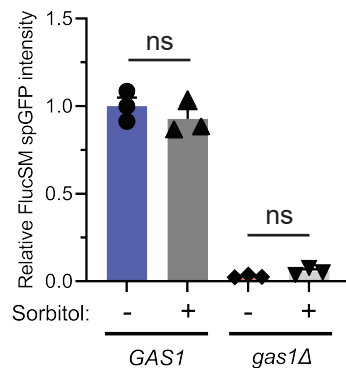

Supplement: jkad263_Supplementary_Data [file jkad263_supplementary_data.zip › Figure_S2_G3-2023-404628.pdf]
